# 656 Burn Injuries at Home: Analyzing the Impact of COVID-19 Lockdowns in Country

**DOI:** 10.1093/jbcr/iraf019.285

**Published:** 2025-04-01

**Authors:** Gabriel García-González, Gerardo Muñoz-Maldonado, Marco Hernández-Guedea, Mauricio Manuel García-Pérez, José Alipi-García

**Affiliations:** Hospital Universitario Dr. José Eleuterio González; Hospital Universitario Dr. José Eleuterio González; Hospital Universitario Dr. José Eleuterio González; Hospital Universitario Dr. José Eleuterio González; Hospital Universitario Dr. José Eleuterio González

## Abstract

**Introduction:**

Burn injuries in a domestic environment are both common and significant, posing a public health issue globally. The epidemiology varies by region, but several consistent patterns emerge from the literature. In low and middle-income countries (LMICs), the burden of burn injuries is exacerbated by multiple factors. The lack of safety regulations and poor appliance design contribute to the high incidence of burns.

**Methods:**

We conducted an analytical cross-sectional study with a digital survey distributed to the COUNTRY population. We chose three of the more populated cities in the country (STATE1, STATE2, and STATE3) and received 1,004 responses. After they provided informed consent, participants were questioned about demographics and the events concerning lesions sustained previously and during the pandemic under the government’s “stay at home” policy.

**Results:**

The collected sample (1,004 participants) was composed of 459 (45.7%) men and 545 (54.3%) women between 18 and 75 years of age, with a mean of 28.7 SD ± 12.94; most of the participants were younger than 30 years of age (66.7%). Most participants had a bachelor’s degree or higher (45.1%).

Participants registered an average of 11.93 (SD ± 4.19) hours spent at home the year before the pandemic and 19.14 (SD ± 4.44) hours during the pandemic, which represents a 60.4% augment on the time spent at home. The frequency of injuries also changed between the year before and the first year of the pandemic: The average numbers of burns were 0.41 SD ± 0.97 and 0.68 SD ± 1.53, respectively, increasing by 66.18% between the 2 years analysed.

As the time spent at home increased a 60.4% (7.21 ± 0.25 h), when compared to wounds per participant, these decreased their incidence by 30.71% in the function of time spent at home. Musculoskeletal injuries also decreased in 45.05% in the function of time spent at home. However, the incidence of burns increased a 5.78% when compared to the increase of time spent at home.

**Conclusions:**

This is the first study in COUNTRY and REGION to identify in-house lesions during the COVID-19 lockdown and to provide useful information for differentiating at-risk populations. From the three types of injuries analyzed, we conclude that, while the risk of wounds and musculoskeletal injuries decreases as people spend more time inside their houses, burn injuries become more frequent. This emphasized the importance of examining house activities, as they shouldn’t be taken as “low risk” just because of the common conception of our homes being a “safe place”.

**Applicability of Research to Practice:**

Domestic burns that doesn’t reach hospital care are usually neglected when taking decisions about policy and society education. This study provides an insight on the reality of burn injuries and how they behave differently than other type of domestic injuries, especially during long periods of time inside the house, like COVID-19 lockdown measures.

**Funding for the Study:**

N/A

